# Coding and non-coding variants in the *SHOX2* gene in patients with early-onset atrial fibrillation

**DOI:** 10.1007/s00395-016-0557-2

**Published:** 2016-04-30

**Authors:** Sandra Hoffmann, Sebastian Clauss, Ina M. Berger, Birgit Weiß, Antonino Montalbano, Ralph Röth, Madeline Bucher, Ina Klier, Reza Wakili, Hervé Seitz, Eric Schulze-Bahr, Hugo A. Katus, Friederike Flachsbart, Almut Nebel, Sabina PW. Guenther, Erik Bagaev, Wolfgang Rottbauer, Stefan Kääb, Steffen Just, Gudrun A. Rappold

**Affiliations:** Department of Human Molecular Genetics, Institute of Human Genetics, University Heidelberg, INF 366, 69120 Heidelberg, Germany; DZHK (German Centre for Cardiovascular Research), Partner site Heidelberg/Mannheim, Heidelberg, Germany; Department of Medicine I, University Hospital Munich, Ludwig-Maximilians-University Munich (LMU), Munich, Germany; DZHK (German Centre for Cardiovascular Research), Partner site Munich, Munich, Germany; Department of Internal Medicine II, University of Ulm, Ulm, Germany; Institut de génétique humaine (CNRS UPR 1142), Montpellier, France; Department of Cardiovascular Medicine, Institute for Genetics of Heart Diseases, University Hospital Münster, Münster, Germany; Department of Internal Medicine III, University Hospital Heidelberg, Heidelberg, Germany; Institute of Clinical Molecular Biology, University of Kiel, Kiel, Germany; Department of Cardiac Surgery, University Hospital Munich, Ludwig-Maximilians-University Munich (LMU), Munich, Germany

**Keywords:** Atrial fibrillation, Cardiac conduction system, MicroRNA, SHOX2, Transcription factor

## Abstract

Atrial fibrillation (AF) is the most prevalent cardiac arrhythmia with a strong genetic component. Molecular pathways involving the homeodomain transcription factor Shox2 control the development and function of the cardiac conduction system in mouse and zebrafish. Here we report the analysis of human *SHOX2* as a potential susceptibility gene for early-onset AF. To identify causal variants and define the underlying mechanisms, results from 378 patients with early-onset AF before the age of 60 years were analyzed and compared to 1870 controls or reference datasets. We identified two missense mutations (p.G81E, p.H283Q), that were predicted as damaging. Transactivation studies using SHOX2 targets and phenotypic rescue experiments in zebrafish demonstrated that the p.H283Q mutation severely affects SHOX2 pacemaker function. We also demonstrate an association between a 3′UTR variant c.*28T>C of *SHOX2* and AF (*p* = 0.00515). Patients carrying this variant present significantly longer PR intervals. Mechanistically, this variant creates a functional binding site for *hsa*-*miR*-*92b*-*5p*. Circulating *hsa*-*miR*-*92b*-*5p* plasma levels were significantly altered in AF patients carrying the 3′UTR variant (*p* = 0.0095). Finally, we demonstrate significantly reduced *SHOX2* expression levels in right atrial appendages of AF patients compared to patients with sinus rhythm. Together, these results suggest a genetic contribution of *SHOX2* in early-onset AF.

**Electronic supplementary material:**

The online version of this article (doi:10.1007/s00395-016-0557-2) contains supplementary material, which is available to authorized users.

## Introduction

Developing approaches towards disease prediction and treatment of heart rhythm disorders is an important topic in healthy aging. Atrial fibrillation (AF) is the most common cardiac rhythm disturbance affecting 1–2 % of the general population [34]. Disorganized electrical activity of the atria results in irregular and often rapid heart rates. Prevalence of AF generally increases with advancing age and the presence of concomitant cardiovascular diseases [34]. Many important aspects of AF pathophysiology have been described [4, 23], and various studies have demonstrated that the genetic background plays a significant role in its pathogenesis [14, 43, 46].

In 2003, Chen et al. published the first report on a mutation in the potassium channel gene *KCNQ1* co-segregating with AF in a single family [9]. Since then, mutations have been identified in various genes encoding ion channels, cardiac gap junctions and signaling molecules. These defective proteins have been shown to contribute to abnormal electrical properties, thereby leading to increased susceptibility of inherited AF [37]. Transcription factors have been recently emerged as important contributors to AF susceptibility [36]. In addition to rare mutations in transcription factor genes with a strong phenotype (*GATA4/5/6, NKX2.5, TBX5)*, common variants located in or close to transcription factor genes (*PITX2, ZFHX3, PRRX1, MEIS1, NKX2.5, TBX5*) have been identified to be associated with AF by genome wide association studies (GWAS) [16, 18, 22, 25, 39, 46]. Genetic variants upstream of the *PITX2* gene (4q25 risk locus) for example, show the strongest association with AF [22, 28], but the SNPs in this region have not been directly linked to expression levels of *PITX2* in patients. Nonetheless, our current understanding of PITX2 function strongly suggests a functional link between this gene and AF. *Pitx2* haploinsufficiency in adult mice results in an increased susceptibility to AF after electrical stimulation [30, 49]. Additional approaches have demonstrated that Pitx2 constitutes a repressor of *Shox2* and thereby inhibits the specification of a left-sided pacemaker, preventing predisposition to AF [49]. More recently it has been shown that a genetic pathway, including *Pitx2*, *miR*-*17*-*92* and *miR*-*106b*-*25* directly repress SAN regulatory genes such as *Shox2,* which delimits SAN development and inhibits AF susceptibility [48]. Similarly, the T-box transcription factor TBX5 which is causative for Holt-Oram syndrome and which in some cases associates with AF, has also been shown to represent an upstream regulator of *SHOX2* [40].

The homeodomain transcription factor Shox2 has various and distinct developmental functions, especially in the development of the sinoatrial node (SAN) region, the primary pacemaker [6, 7, 19, 52]. A knockout mouse model verified this key role for Shox2 in SAN development and specification during early cardiac formation [6, 19]. Homozygous *Shox2*^−*/*−^ embryos die between E11.5 and E17.5 due to cardiovascular defects [6]. This pivotal role in pacemaker function was further demonstrated by the severe bradycardia after knockdown of Shox2 in zebrafish embryos and markedly reduced heart rates in isolated murine *Shox2*^−*/*−^ hearts [6, 19, 24, 32, 40]. Furthermore, it has also been demonstrated that a Shox2 dependent genetic program primes the pacemaker cells in the pulmonary vein myocardium, a vulnerable substrate for ectopic electrical activity initiating AF [53].

Several clinical studies as well as animal experiments have demonstrated a link between sinus node dysfunction and atrial fibrillation; SAN electrical activity, for example, has been demonstrated to modulate pulmonary vein arrhythmogenesis as a trigger of AF [8]. Nonetheless, it is not yet clear whether AF develops as a result of SAN dysfunction or vice versa, or if both share a common pathophysiological mechanism.

In this study, we investigate *SHOX2* as a potential susceptibility gene for atrial fibrillation in a large set of patients with early-onset AF. To identify causal variants and the underlying mechanisms by which they act, we included all coding exons but also parts of the 5′ and 3′ untranslated regions (UTRs) of the *SHOX2* gene. To elucidate the molecular mechanisms, functional in vitro and in vivo studies were carried out.

## Results

### Mutation analysis of the *SHOX2* gene in patients with atrial fibrillation

**Fig. 1:**
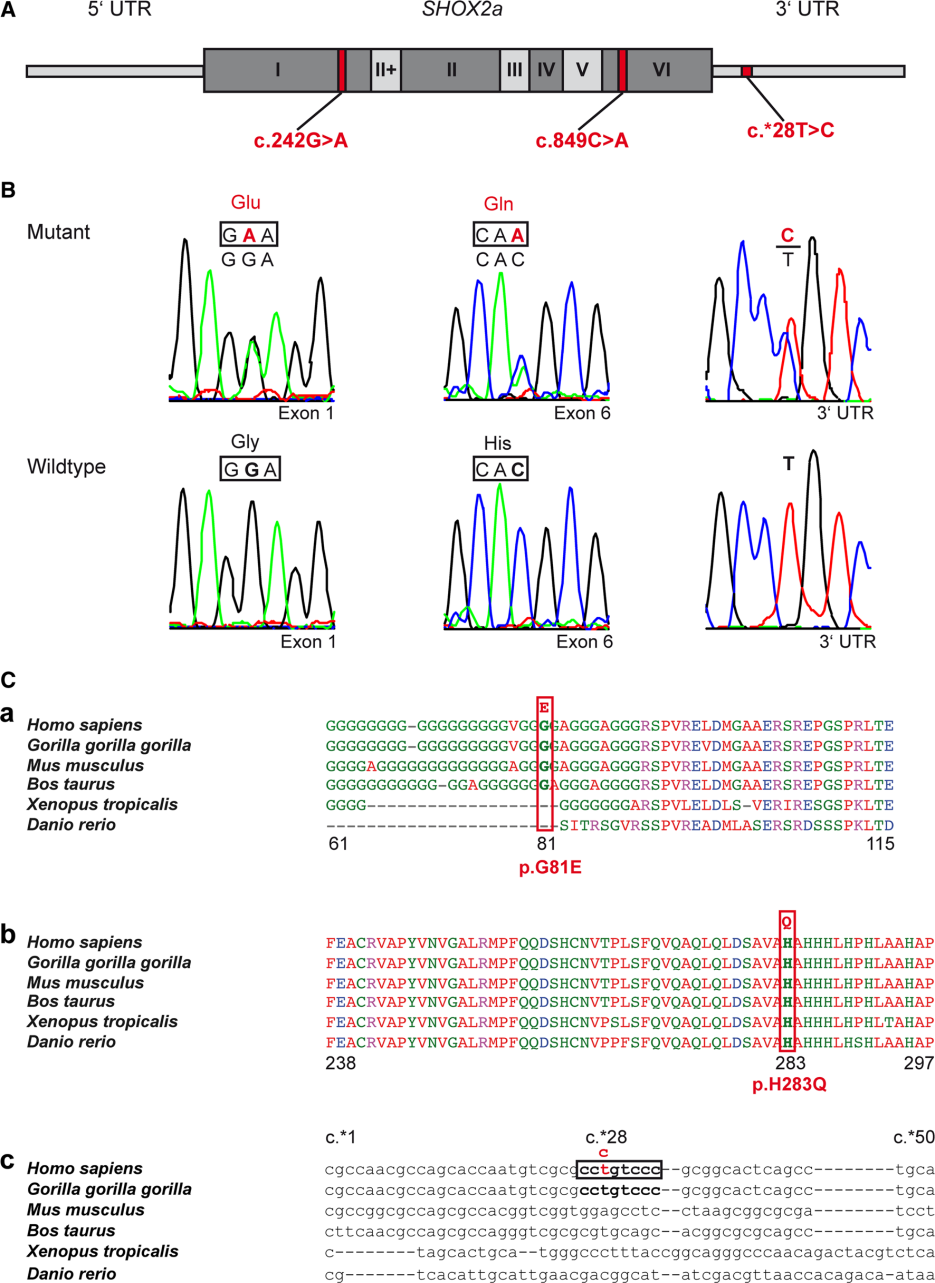
Identified *SHOX2* variants in patients with atrial fibrillation. **A** Schematic drawing showing the position of the identified coding and non-coding variants within the *SHOX2* gene. The *SHOX2a* isoform is composed of 7 exons. All exons are highly conserved between species except exon II^+^ which is restricted to primates. **B** Electropherograms showing the substitutions detected in the *SHOX2* gene in patients with atrial fibrillation and their respective wild type counterparts. **C** Multiple sequence alignment of SHOX2 protein and 3′UTR parts among different species. The amino acid p.G81 encoded within exon 1 is conserved among mammals (*a*). The amino acid p.H283 encoded within exon 6 is highly evolutionary conserved across vertebrates (*b*). The *SHOX2* 3′UTR sequence around the c.*28T>C variant is only present in primates and not conserved between species (*c*). The multiple sequence alignment was done using Clustal Omega for protein sequences and MAFFT program with Q-INS-i algorithm for 3′UTR sequences

To investigate a possible role of *SHOX2* in atrial fibrillation (AF), we performed a mutational screen in 378 patients with early-onset AF before the age of 60 years (14–60 years). Clinical characteristics of the study cohort are listed in Table S1. Sequencing all coding exons as well as parts of the 5′ and 3′UTRs of the *SHOX2* gene identified a variant in the 3′UTR (c.*28T>C; rs138912749) and two missense mutations (c.242G>A, c.849C>A) (Fig. 1A, B).

**Fig. 2:**
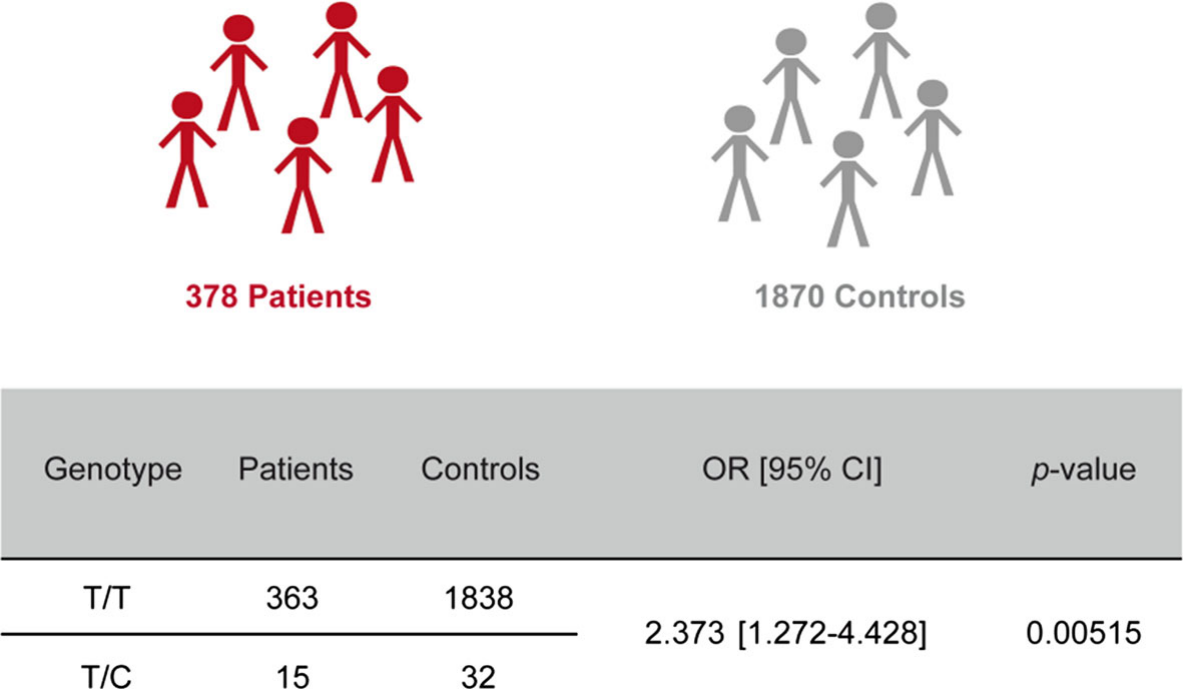
Association analysis of *SHOX2* 3′UTR variant c.*28T>C with atrial fibrillation. Values indicate number of patients and controls with the respective genotype (T/T = wild type; T/C = variant). Odds ratio (OR), 95 % confidence interval (CI) and *p*-value were calculated using the Chi squared (*χ* ^2^) test. The control cohort consists of 294 unrelated healthy individuals and 1576 long-lived individuals from the German longevity collection [38]

The c.*28T>C 3′UTR variant was identified in 15 unrelated individuals. To address the significance of the 3′UTR variant, it was genotyped in a control cohort. Selecting appropriate controls is a particular challenge in case–control association studies. We selected a specific control group with a certain guarantee neither to suffer nor to develop arrhythmias. This group comprised 1576 unrelated long-lived individuals with an age of 95–109 years from the German longevity collection [38]. A second control group matched the patient population in terms of age (25–70 years). Genotyping of a total of 378 unrelated AF patients versus 1576 long-lived controls as well as 294 younger healthy individuals demonstrated a significant association between the *SHOX2* 3′UTR variant and AF (*p* = 0.00515, OR = 2.373) (Fig. 2). Note that both control groups show similar minor allele frequencies (MAF) of 0.9 and 0.7 %, respectively.

The identified missense mutations are both unique and have been detected each in a single patient. The c.242G>A exchange causes an amino acid substitution of glycine to glutamic acid (p.G81E). The c.849C>A mutation results in a substitution of histidine to glutamine (p.H283Q) (Fig. 1B, C). Both amino acids reside at evolutionary conserved sites with p.G81 being conserved among mammals and p.H283 highly conserved among vertebrates (Fig. 1C). Different in silico analyses (PolyPhen2, Mutation Taster, PROVEAN, Hansa) predicted that the p.H283Q missense mutation is disease causing (deleterious), while p.G81E is predicted as possibly damaging. Both SHOX2 amino acid variants were absent in the 1000 Genomes dataset (http://www.1000genomes.org/) and the Exome Variant Server (http://evs.gs.washingon.edu/EVS/). Thus, both mutations were considered as disease causing and further investigated.

### Functional characterization of the *SHOX2* 3′UTR variant

**Fig. 3:**
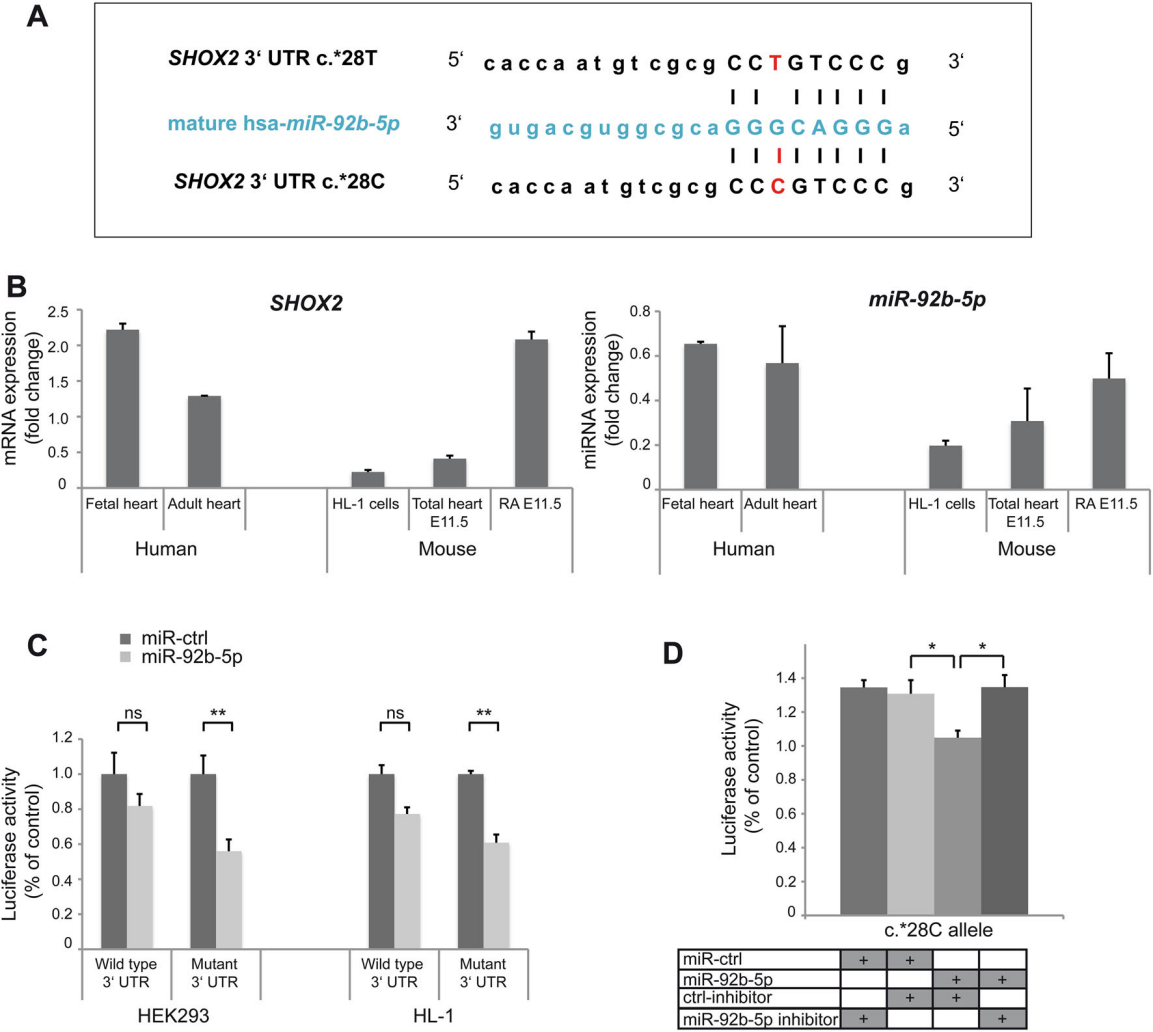
Functional characterization of *SHOX2* 3′UTR variant. **A** *SHOX2* 3′UTR with the c.*28C allele creates a novel *miR*-*92b*-*5p* binding site (pairing of the seed region is shown). **B** *SHOX2* mRNA (*left*) and *miR*-*92b*-*5p* (*right*) expression levels in human and murine heart tissues (human fetal and adult heart, mouse embryonic total heart and right atrium (RA) E11.5) and HL-1 cells (derived from mouse atrial cardiomyocytes). *SHOX2* mRNA and miRNA (*miR*-*92* *b*-*5p*) are co-expressed in the same tissues. **C** Luciferase activity of wild type (c.*28T) or mutant (c.*28C) psiCHECK2-*SHOX2* 3′UTR constructs, co-expressed with *miR*-*92b*-*5p* (indicated in *light grey*) or negative control miRNA (miR-ctrl, indicated in *dark grey*) in HEK293 and HL-1 cells, determined 48 h after transfection. *miR*-*92b*-*5p* significantly reduces the activity of the mutant but not the wild type *SHOX2* 3′UTR reporter in both cell lines. **D** HEK293 cells were transiently co-transfected with mutant (c.*28C) psiCHECK2-*SHOX2* 3′UTR reporter and *miR*-*92b*-*5p* together with either *miR*-*92b*-*5p* inhibitor or negative control inhibitor (ctrl-inhibitor) and luciferase activity was determined after 48 h. The allele-specific effect of *miR*-*92b*-*5p* on the luciferase activity of the mutant *SHOX2* 3′UTR reporter is reversed by a specific *miR*-*92b*-*5p* inhibitor, but not altered using the control inhibitor. Data are expressed as mean ± SEM of 3–4 independent experiments. For each experiment triplicates were measured. *p* values were determined by a paired *t* test (*p < 0.05; **p < 0.01, *ns* not significant)

To functionally characterize the 3′UTR variant exhaustive search for 6-mer seed matches (i.e. perfect complement to miRNA nt 2-7) to the known human miRNAs (as in miRBase v20; http://www.mirbase.org/) was carried out and revealed that the c.*28T>C variant in the 3′UTR creates a putative 8 base binding site for *hsa*-*miR*-*92b*-*5p* in the *SHOX2* gene (Fig. 3A). Co-expression of mRNA and miRNA is a key requirement for a regulatory mechanism. Expression of *hsa*-*miR*-*92b*-*5p* was therefore first tested in various fetal and adult human tissues including heart (Fig. S1). Comparative expression analysis of *SHOX2* and *miR*-*92b*-*5p* was then carried out in human fetal and adult heart, mouse E11.5 right atrium (containing the SAN region) and total heart, and murine HL-1 cells. Both, mRNA and miRNA are co-expressed in these tissues (Fig. 3B). *SHOX2* expression has also been previously demonstrated in the SAN of human embryos (7 weeks post-conception), implicating a similar role of human and mouse *SHOX2* in SAN development [31].

To assess the functional relevance of the novel *miR*-*92b*-*5p* binding site, we carried out luciferase reporter assays. Reporter constructs containing the full-length wild type (c.*28T) or mutant (c.*28C) *SHOX2* 3′UTRs were generated. Transient co-transfection of HEK293 cells and atrial cardiomyocytes (HL-1 cells) with wild type or mutant reporter constructs together with *miR*-*92b*-*5p* or negative control miRNA revealed significantly reduced luciferase activity of the mutant. Both cell lines revealed similar results, HEK293: *p* = 0.0070; HL-1: *p* = 0.0052 (Fig. 3C). Comparable results were obtained using a second luciferase reporter vector (pRL-TK) containing only part of the *SHOX2* 3′UTR (Fig. S2). To confirm the specificity of *miR*-*92b*-*5p* on luciferase activity of the mutant *SHOX2* 3′UTR reporter, we repeated the assays using a specific *miR*-*92b*-*5p* or negative control inhibitor. While the allele-specific effect of *miR*-*92b*-*5p* was completely reversed by the *miR*-*92b*-*5p* inhibitor, it was not altered using a control inhibitor (Fig. 3D). Together, these results show that *miR*-*92b*-*5p* is capable of targeting the *SHOX2* 3′UTR carrying the c.*28C variant.

**Fig. 4:**
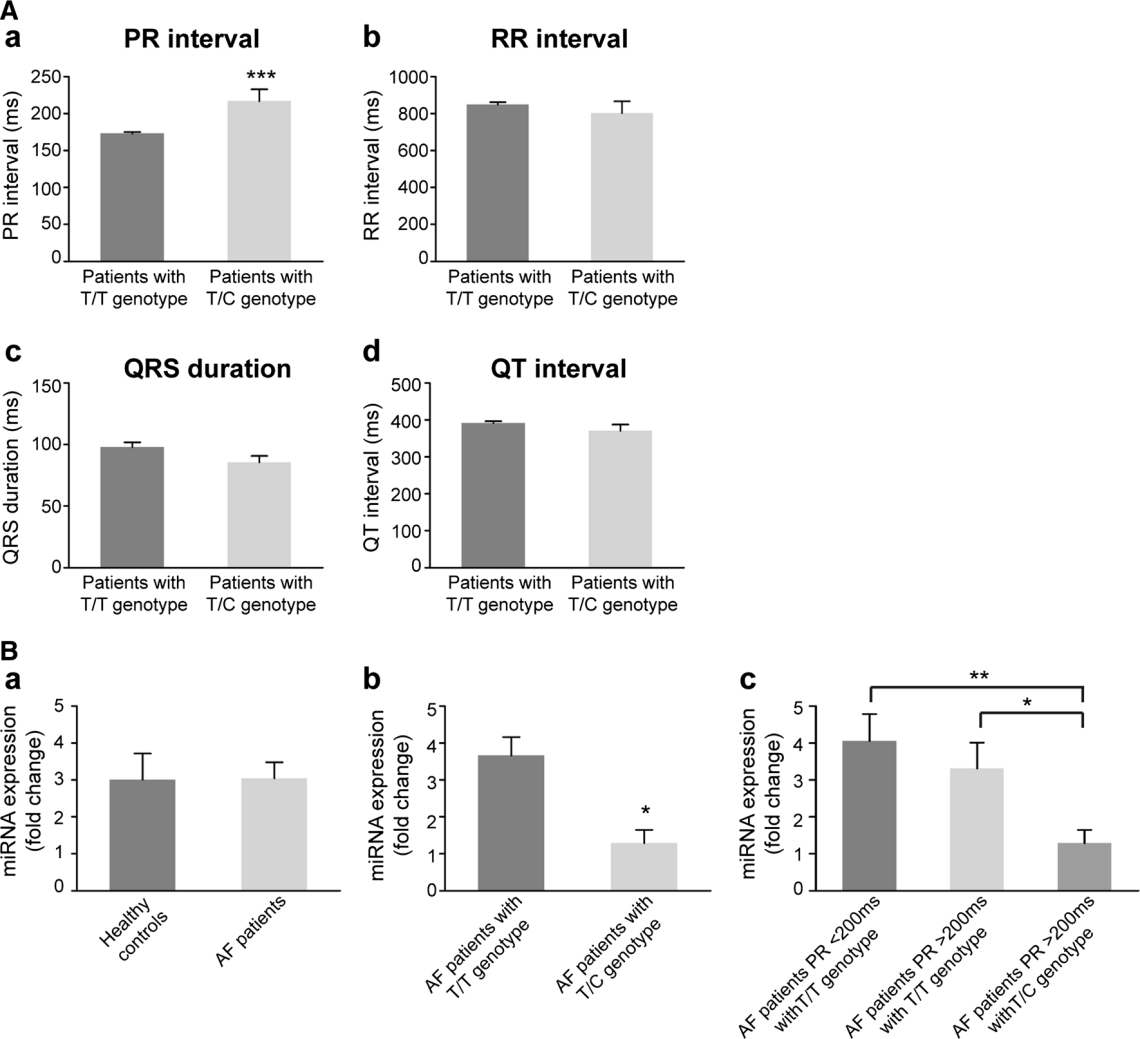
Clinical data analysis of AF patients. **A** Electrocardiographic parameters of analyzed AF patients. Patients not carrying the *SHOX2* 3′UTR variant c.*28T > C (*dark grey bars*, *n* = 361, T/T genotype) compared to carriers of the variant (*light grey bars*, *n* = 15, T/C genotype). PR interval (*a*). RR interval (*b*). QRS duration (*c*). QT interval (*d*). Data are presented as mean ± SEM. *p*-values were determined by an unpaired *t* test (***p < 0.001). **B** *MiR*-*92b*-*5p* expression in human plasma. Healthy controls (*n* = 12) compared to AF patients (*n* = 23) (*a*). AF patients with T/T genotype (*n* = 17) compared to AF patients with T/C genotype (*n* = 6) (*b*). AF patients with T/C genotype and PR intervals >200 ms (*n* = 6) compared to AF patients with T/T genotype and PR intervals >200 ms (*n* = 9) or to AF patients with T/T genotype and PR intervals <200 ms (*n* = 8) (*c*). Data are presented as mean ± SEM. *p*-values were determined by an unpaired *t* test (*p < 0.05; **p < 0.01)

Interestingly, subsequent detailed clinical data analysis of patients carrying the 3′UTR variant (T/C genotype) revealed a significantly prolonged PR interval over 200 ms reflecting prolonged atrial and atrioventricular node conduction (Fig. 4Aa, Table S1) and indicating alterations of either the sinoatrial, atrial, or atrioventricular nodal electrical properties. No differences were seen for other electrocardiographic (ECG) parameters (RR interval, QRS duration, QT interval) or any clinical characteristics (age at onset, type of AF, family history, concomitant diseases, risk factors, or echocardiographic parameters) comparing AF patients carrying the variant (T/C) to patients with the T/T genotype (Fig. 4Ab-d, Table S1).

As changes in circulating miRNA levels often reflect an altered physiological state of an organ or tissue [13], we investigated the expression of *miR*-*92b*-*5p* in the plasma of AF patients versus healthy controls. No significant differences were detected (Fig. 4Ba). Clinical characteristics of patients where plasma samples were available are listed in Table S2. When we, however, split the patient cohort in T/T (*n* = 17) and T/C (*n* = 6) genotype carriers, patients with the T/C genotype had significantly decreased *hsa*-*miR*-*92b*-*5p* plasma levels (*p* = 0.0129) compared to AF patients with T/T genotype (Fig. 4Bb). When we further split the AF patients with T/T genotype into two groups according to their PR interval length (<200 ms, *n* = 8; >200 ms, *n* = 9) and compare them with T/C genotype carriers with prolonged PR intervals (*n* = 6), the effect is even more pronounced (*p* = 0.0095) (Fig. 4Bc).

These findings point to *hsa*-*miR*-*92b*-*5p* as a putative regulator of *SHOX2* expression in a distinct subgroup of AF patients.

### Functional relevance of *SHOX2* missense mutations

**Fig. 5:**
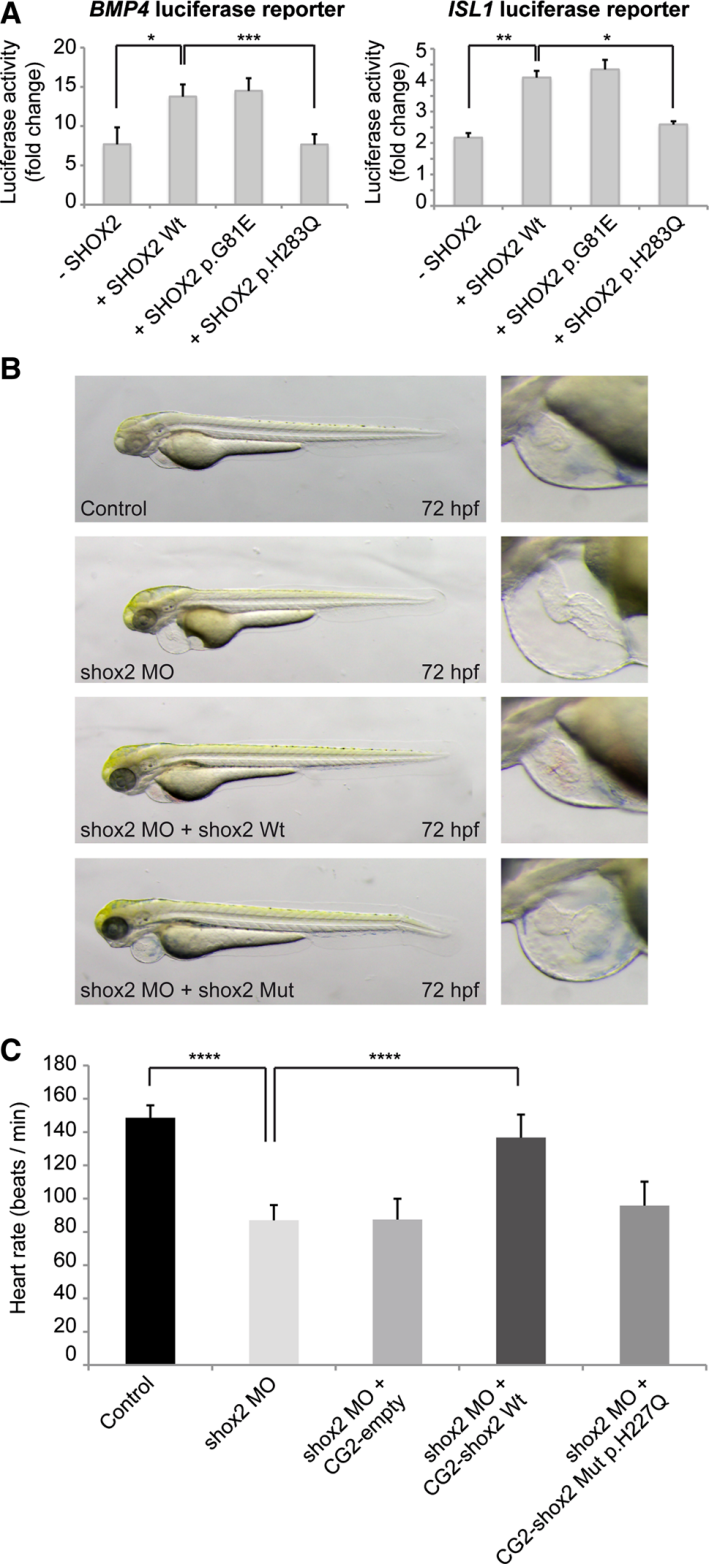
Functional characterization of the *SHOX2* coding variants. **A** Luciferase activity of *BMP4* (*left*) or *ISL1* (*right*) luciferase reporter constructs co-expressed with SHOX2 wild type (Wt) or SHOX2 mutants (p.G81E; p.H283Q) in HEK293 cells (human embryonic kidney cells), determined 24 h after transfection. All values are reported as fold changes of luciferase activity normalized to the empty vector (pGL3 basic) co-transfected with the respective expression constructs. Data are expressed as mean ± SEM of four independent experiments performed in triplicate. *p* values were determined by a paired *t* test (*p < 0.05; **p < 0.01; ***p < 0.001). **B** Injection of *shox2* antisense morpholino (MO), which leads to pericardial edema and pericardial blood congestion due to reduced heart rates in zebrafish can not be rescued by cardiomyocyte-specific overexpression of shox2 Mut p.H227Q (corresponds to human SHOX2 p.H283Q mutation) compared to shox2 Wt (wild type) 72 h post fertilization (hpf). **C** The mean heart rate of control embryos 72 hpf amounts to 149 beats per minute (bpm). After shox2 knockdown, the heart rate is significantly reduced to 87 bpm. While cardiomyocyte-specific overexpression of Wt shox2 rescues the MO-mediated bradycardia (137 bpm), overexpression of shox2 Mut p.H227Q does not alter the reduced heart rate significantly at 72 hpf (95 bpm). Data are expressed as mean ± SEM of 3 independent experiments. *p* values were determined by one-way ANOVA with Sidak’s multiple comparisons test (****p < 0.0001; *ns* not significant; *n* = 10–16 per condition per experiment)

To investigate the functional significance of the two missense mutations, subcellular localization of the SHOX2 mutants was determined in HEK293 and HL-1 cells and compared to wild type. Consistent with its role as transcription factor, wild type as well as p.G81E or p.H283Q SHOX2 mutants localized to the nucleus (data not shown). Next, we performed luciferase reporter assays to determine whether the identified mutations have an impact on the transactivational activity of SHOX2. Wild type SHOX2 activates the promoter of its previously described target genes *BMP4* and *ISL1* [24, 40]. We could show that SHOX2 harboring the p.G81E mutation has still transactivational activity similar to wild type, while the p.H283Q mutant severely affects the transactivational activity and was not able to activate its targets (Fig. 5A).

Functional in vivo characterization was carried out in zebrafish for the p.H283Q mutation, which affects an evolutionary highly conserved amino acid (the p.G81E mutant was not conserved in zebrafish). DNA constructs expressing wild type and mutant *shox2* under the control of the *cardiac myosin light chain 2* (*cmlc2*) promoter [26], driving myocardial-specific expression, were used in overexpression experiments (zebrafish Shox2 p.H227Q corresponds to human SHOX2 p.H283Q). The *cmlc2* promoter also drives expression of GFP, allowing the monitoring of cardiomyocyte-specific expression. The mean heart rate of control embryos 72 h post fertilization (hpf) amounts to 150 beats per minute (bpm).

Injection of the *shox2* mutant p.H227Q expression construct into wild type zebrafish embryos at the one-cell stage did not cause any changes in embryonic heart rate (151 bpm) at 72 hpf, implying that the Shox2 p.H227Q mutant does not act in a dominant-negative manner in zebrafish in vivo.

As shown previously, targeted knockdown of zebrafish Shox2 using Morpholino-modified antisense oligonucleotides (MO) leads to pericardial edema and pericardial blood congestion due to severely reduced heart rates in zebrafish embryos [24]. We also demonstrated that bradycardia in Shox2 morphants can be rescued by cardiomyocyte-specific expression of wild type Shox2 [24]. Here, we used this approach to address whether the Shox2 mutant is also able to rescue the Shox2 morphant phenotype or whether the p.H227Q mutation leads to a loss of Shox2 function. First we co-injected 0.32 ng of the respective expression construct along with 2.8 ng *shox2* MO, sufficient to induce the known bradycardia phenotype [24] and then monitored visible morphological changes and heart rate of GFP positive embryos after 72 hpf. While myocardial-specific expression of wild type Shox2 could rescue the pericardial edema and reduced heart rate in Shox2 morphant embryos (137 vs. 87 bpm; *p* = <0.0001), this was not the case for the p.H227Q mutant (96 vs. 87 bpm) (Fig. 5B, C). These data indicate that the zebrafish p.H227Q mutation (corresponding to human p.H283Q) leads to a loss of regular Shox2 function and thereby impairs cardiac pacemaker function in vivo.

### *SHOX2* expression in right atrial appendages of AF patients

**Fig. 6:**
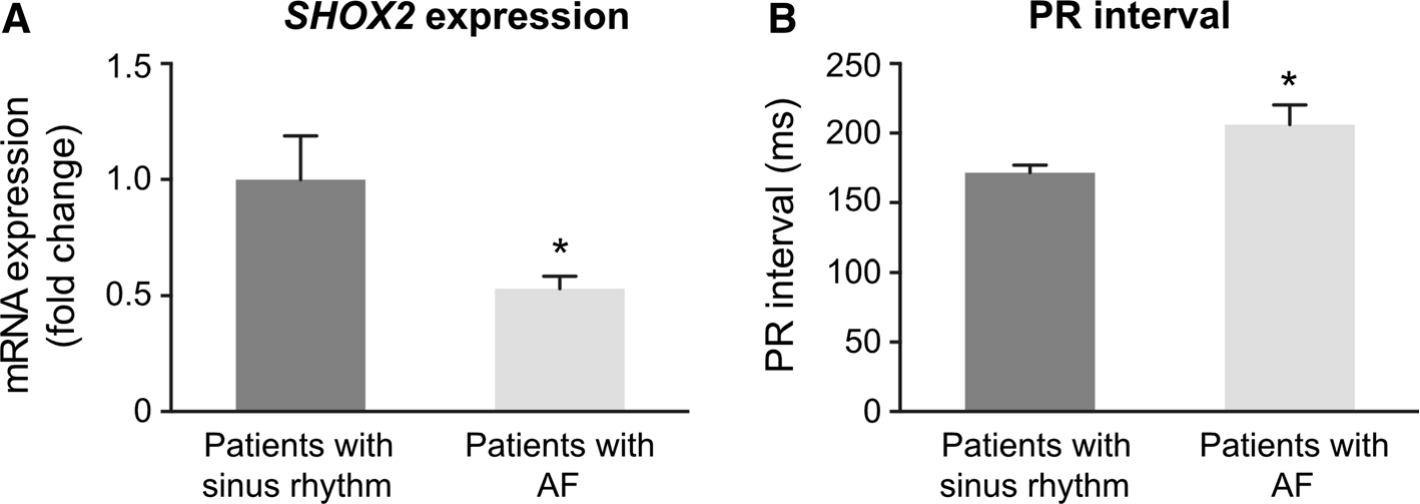
*SHOX2* expression in right atrial tissue of AF patients. **A** *SHOX2* mRNA levels are significantly reduced in right atrial appendages from patients with AF (*n* = 17) compared to patients with sinus rhythm and no previous history of AF (*n* = 17). **B** PR intervals (where available) are significantly prolonged in AF subjects (*n* = 10) compared to patients with sinus rhythm (*n* = 15). Data are presented as mean ± SEM. *p* values were determined by an unpaired *t* test (*p < 0.05)

To investigate *SHOX2* expression levels in human tissue specimens, right atrial appendages were obtained during cardiac surgery from patients with AF and patients with sinus rhythm (*n* = 17 for each group). Expression analysis revealed significantly reduced *SHOX2* transcripts in AF patients compared to the control group (*p* = 0.0226; Fig. 6A). Next, we examined ECG recordings from these patients and found significantly longer PR intervals (*p* = 0.0157) in AF patients compared to patients with sinus rhythm (Fig. 6B), whereas other ECG parameters (RR interval, QRS duration, QT interval) were unchanged (data not shown).

Together, our data demonstrate for the first time coding and non-coding variants in *SHOX2* in patients with atrial fibrillation. We also established a link between the observed genotype to an intermediate AF phenotype with prolonged PR interval inferred from reduced *SHOX2* expression levels in this cohort.

## Discussion

Genetic causes underlying cardiac arrhythmias are many-fold and incompletely understood. Gaining more insights into the mechanistic basis of atrial fibrillation (AF), the most common arrhythmia, will allow the development of more effective, personalized diagnostic and therapeutic approaches. In the last few years, transcription factors, their targets and transcriptional modifiers have been shown to play important roles in AF pathophysiology, indicating that dysregulated gene expression is an essential underlying mechanism [36].

Previous studies in mouse and zebrafish have suggested a pivotal role for the transcription factor Shox2 during cardiac development, especially in the pacemaker region and sinoatrial node (SAN) function, suggesting *SHOX2* as possible susceptibility gene for cardiac arrhythmias [6, 24, 32, 40]. Our current study provides first convincing evidence for a link between *SHOX2* and atrial fibrillation in humans. Our study cohort comprised 378 AF patients with an early-onset of disease before the age of 60 years. Screening this patient cohort, three different heterozygous sequence variants were identified, including a 3′UTR variant and two novel missense mutations.

The 3′UTR variant c.*28T>C was identified in 15/378 patients (MAF 2 %). It was also found to be present in the general population (MAF 0.7–0.9 %) without known AF. In the control group comprising 1576 German long-lived individuals (above 95 years of age), the c.*28T>C variant was detected with a MAF of 0.9 %. Additional genotyping of 294 unrelated healthy German individuals (25–70 years) revealed a MAF of 0.7 %. Statistical validation of the 3′UTR variant in 378 patients versus the combined control cohorts (1870 individuals) resulted in a statistical significance of *p* = 0.00515. Public databases (1000 Genomes Project), annotated this variant as a SNP (rs138912749) with a MAF of 0.7 %, comparable to our genotyped control cohorts.

The majority of miRNAs recognize their targets solely through direct binding to their seed sequence. Regulatory variants in the 3′UTR of genes may therefore create or disturb miRNA binding sites. So far only a few examples of regulatory variants have been identified and linked to a disease or phenotype [1, 3, 12, 29]. We have identified a SNP in the 3´UTR of *SHOX2* that has been shown to create a novel miRNA target site for *miR*-*92b*-*5p*. MiRNAs represent important post-transcriptional modifiers of gene expression by targeting the 3′UTR of messenger RNAs and thereby controlling developmental processes [5, 33]. Collectively, they affect nearly all cellular pathways including proarrhythmogenic signaling cascades in the heart [41, 47, 50]. Several miRNAs have been demonstrated in the pathophysiology of AF. Either they are involved in the control of atrial electrical remodeling by regulating ion channels, transporters and Ca^2+^-handling proteins, or have an impact on atrial structural remodeling by regulating fibrosis and apoptosis [15, 35, 41]. Both, electrical and structural remodeling processes of the atria are strongly associated with AF. Recently, it has also been shown that Pitx2 upregulates the miR-17-92/106b-25 cluster, that in turn represses Shox2 resulting in delimited sinoatrial node development. Deletion of this miRNA cluster in mice results in sinus node dysfunction, PR prolongation and predisposition of AF [48]. This study demonstrates that Shox2 is an important factor regulating the development and maintenance of the whole cardiac conduction system rather than exclusively mediating sinus node development.

The two miRNAs, *miR*-*92b*-*3p* and *92b*-*5p,* are processed from the same precursor molecule, but only the biological function of *miR*-*92b*-*3p* has been investigated so far [10, 54]. *Hsa*-*miR*-*92b*-*3p* has been implicated in various cardiovascular disorders including coronary artery disease and chronic systolic heart failure [21, 45]. Here, we provide first evidence for a functional relevance of *miR*-*92b*-*5p* during heart development and in the pathogenesis of AF. We demonstrated that a novel *hsa*-*miR*-*92b*-*5p* target site generated by a genetic variant in the 3′UTR of the *SHOX2* gene may reduce *SHOX2* expression and thereby facilitate proarrythmogenic remodeling leading to AF. This appears to have a remarkable effect on circulating *hsa*-*miR*-*92b*-*5p* plasma levels, which are decreased by threefold in those patients with the T/C genotype (*n* = 6), compared to AF patients with the wild type allele (*n* = 8). Although miRNA levels in plasma could be investigated only in a limited number of patients so far, a significant difference was noted.

A deeper understanding of the reciprocal function of miRNAs and their targets according to their relative abundance inside the cell is still lacking. As a mechanism one may argue that altered intracellular *miR*-*92b*-*5p* target site abundance may result in decreased extracellular levels, since more *miR*-*92b*-*5p* is used within the tissue. This option, however, seems unlikely, as for most mRNAs its contribution to miRNA binding is largely diluted by the effects of all the other mRNA in the cell [17]. One could also hypothesize that some targets are known to induce miRNA degradation leading to decreased extracellular plasma levels [2] but so far little is known about the mechanisms or the determinants of this phenomenon.

We have found that the AF patients carrying the *SHOX2* 3′UTR c.*28C allele have significantly prolonged PR intervals compared to patients with the wild type allele. Results of the Framingham heart study demonstrated that prolonged PR intervals increase the risk for AF and predict a more severe disease progression and worse prognosis [11]. Furthermore, our finding is in line with the study by Wang et al. showing that miRNA-mediated alterations of *Shox2* expression in mice also result in PR prolongation and increased susceptibility to AF [48].

Cardiogenesis is a developmental process tightly controlled by an orchestrated network of transcriptional regulators. Correct dosage is essential, since haploinsufficiency of a single regulatory factor can shift a fine-tuned balance and result in severe defects [42]. In the mouse, the homozygous loss of Shox2 results in embryonic lethality with bradycardia, whereas heterozygous mice have not yet been studied in much detail [6, 19]. In a mouse model where *Shox2* was replaced by the closely related but hypomorphic *SHOX* allele, embryonic lethality could be rescued, but arrhythmias remained [31]. These data add further weight to the findings that haploinsufficient mutations in *SHOX2* can trigger arrhythmia phenotypes, suggesting a maintenance role of SHOX2 in the adult heart.

To understand the underlying molecular mechanism by which the identified missense mutations may act, transactivation studies with the two known direct SHOX2 target genes *BMP4* and *ISL1* [40] were carried out. While the p.G81E mutant did not affect the transactivational activity of SHOX2, the p.H283Q mutant was unable to activate both *BMP4* and *ISL1* target genes. Further functional validation of the p.H238Q mutant was then carried out in zebrafish. Cardiac electrophysiology in the zebrafish displays several common features compared to humans. First, the heart rate of an adult zebrafish (about 120 bpm) is very similar to the human situation. Excitation is generated in specialized pacemaker cells at the inflow tract of the atrium [52] and is rapidly propagated to the ventricle with a characteristic delay at the atrioventricular canal. Interestingly, cardiac pacemaker cells closely resemble those in mammals especially in respect to their gene expression pattern and electrochemical properties [52]. Furthermore, the cellular and molecular mechanisms that underly automaticity, refractoriness and conduction of the heart are conserved between zebrafish and humans [44, 46].

It was previously demonstrated that MO-mediated shox2 deficiency in zebrafish leads to pericardial edema and blood congestion due to severely reduced heart rates [6, 24]. Using this phenotype as a read out, we injected the zebrafish p.H227Q mutant (which corresponds to the human p.H283Q mutation) in a rescue experiment. In contrast to wild type, the mutant Shox2 was not able to compensate the Shox2 deficiency and rescue the phenotype. These data demonstrate that the missense mutation H283Q, which affects a highly conserved amino acid, severely impairs the biological function of SHOX2.

In summary, several studies have demonstrated that Shox2 is an important regulator of the cardiac conduction system and that alterations of Shox2 expression can impair electrical properties of the heart resulting in sinus node dysfunction, conduction slowing, or arrhythmias [6, 52]. To determine if these experimental findings also hold true in humans, *SHOX2* expression was measured in heart tissue from patients. Significantly reduced *SHOX2* expression levels were demonstrated in atrial tissue from patients with AF compared to patients with normal sinus rhythm and no previous history of AF. These findings are hypothesis-generating and we propose that reduced *SHOX2* expression caused by coding and regulatory variants in patients contribute to the observed arrhythmic phenotype of AF with prolonged PR interval.

Developing a mechanistic classification of AF based on health modifiers like genetic susceptibility is essential to improve personalized prevention and management of AF [20]. Our findings contribute to a better classification of AF, since a specific patient cohort with early-onset AF and prolonged PR interval could be determined by genetic predisposition involving the *SHOX2* gene.

Our findings have also provided genetic evidence for a novel miRNA target site contributing to and resulting in AF predisposition. Replication in other large patient cohorts will show, whether circulating *miR*-*92b*-*5p* has the potential to serve as a non-invasive biomarker and promising therapeutic target in the personalized diagnosis, prevention and treatment of AF.

## Materials and methods

### Study sample

#### Patient cohort

The patient cohort comprised 378 German individuals with atrial fibrillation (AF). Only patients with early-onset AF were included, i.e. onset of AF before the age of 60 years (14–60 years). Patients were recruited from 2001 to 2013 in the context of a general biobanking effort. About 80 % of all eligible patients were recruited; around 55 % of the patient samples have been previously used in other studies. AF cases were recruited from the Department of Medicine I of the Ludwig-Maximilians-University Hospital Grosshadern, Munich. Detailed patient characteristics are listed in Tables S1 and S2. Clinical parameters were available for at least 95 % of all cases.

#### Control cohort

For follow-up genotyping of the *SHOX2* c.*28T>C variant, we investigated 1576 unrelated long-lived individuals with an age between 95 and 109 years who were recruited from across Germany. A detailed description of the sample and the recruitment procedure is reported elsewhere [38]. A second control population consisted of 294 ethnically matched unrelated healthy individuals (25–70 years), recruited from the Institute of Human Genetics of the University Hospital Heidelberg. As healthy controls for the miRNA measurement, we used plasma samples from age-matched volunteers without history of cardiac disease.

### *SHOX2* expression in human tissue samples

Human right atrial appendages (RAA) were collected from patients undergoing cardiac surgery at the Department for Cardiothoracic Surgery at Ludwig-Maximilians-University Hospital Grosshadern, Munich. Seventeen patients with AF and 17 patients with sinus rhythm and no previous history of AF were recruited from 2014 to 2015. After excision tissue was snap-frozen in liquid nitrogen and stored at −80 °C. Total RNA was isolated using TRIzol. cDNA was synthesized using the SuperScript First-Strand Synthesis System for RT-PCR (Invitrogen). *SHOX2* expression was measured by qPCR using TaqMan probes for *SHOX2* and *GAPDH* as a housekeeping gene (Table S3). qPCR was performed using a CFX 96 C1000 touch Realtime Cycler (Bio Rad, Germany). Samples were measured as triplicates.

#### Ethical statement

The study was approved by the local ethics committees and was performed in accordance with the ethical standards laid down in the 1964 Declaration of Helsinki and its later amendments. All probands gave written informed consent including consent to use DNA for genetic analyses prior to their inclusion in the study.

### Sequencing and genotyping

PCR primers used to amplify each exon of *SHOX2*, the flanking intron–exon boundaries and parts of the UTRs are listed in Table S3. We analyzed the longest isoform of *SHOX2* including a primate specific exon 2 + (NM_003030). PCRs were performed with Paq5000 polymerase (from Stratagene) using standard conditions. The PCR products were analyzed by gel electrophoresis. Adequate amplification products were selected for purification and sequencing done by GATC Biotech (GATC Biotech AG, Konstanz, Germany).

Genotyping of the *SHOX2* 3′UTR variant c.*28T>C in long-lived individuals was performed with a Custom TaqMan SNP Genotyping Assay (Life Technologies).

### Generation of plasmid constructs

To introduce the c.242G>A/p.G81E and c.849C>A/p.H283Q mutations into a previously described *SHOX2* expression plasmid (human *SHOX2* cDNA sub-cloned into a pDEST27 vector, Invitrogen [24]) for luciferase reporter assays, site-directed mutagenesis was performed using mutated *SHOX2* primers (Table S3). The zebrafish shox2 expression plasmid harbouring the p.H227Q (corresponding to human p.H283Q) mutation was generated by site directed mutagenesis of the previously described pDestTol2CG2-shox2 plasmid [24] using specific primers (Table S3). To generate luciferase reporter constructs, the *SHOX2* 3′UTR full-length wild type sequence (+1203/+3222) was cloned into the psiCHECK-2 vector (Promega), while a part of the *SHOX2* 3′UTR wild type sequence (+1208/+2185) was cloned into the pRL-TK vector (Promega), both downstream of a Renilla luciferase reporter gene. Site-directed mutagenesis was performed to introduce the c.*28C allele using mutated *SHOX2* 3′UTR primers (Table S3). Site-directed mutagenesis was performed with the QuikChange II Site-Directed Mutagenesis Kit (Agilent Technology) according to manufacture instructions. All constructs were verified by sequencing.

### Cell culture, transfection and luciferase assay

HEK293 cells were cultured at 37 °C in DMEM medium containing high glucose, supplemented with 10 % fetal calf serum and antibiotics. HL-1 cells were cultured at 37 °C in Claycomb medium (Sigma-Aldrich), supplemented with 10 % fetal bovine serum, 2 mM l-glutamine, 100 µM norepinephrine, 10U/ml penicillin and 100 µg/ml streptomycin on flasks pre-coated with gelatine-fibronectin. For luciferase assays, published SHOX2 targets *BMP4* and *ISL1* were used [24, 40]. HEK293 cells were co-transfected with the respective pGL3-basic reporter constructs (1 µg) together with *SHOX2* wild type or mutant expression constructs (1 µg) using PEI. 24 h after transfection, luciferase activity was determined and normalized to Renilla luciferase activity with a dual luciferase assay kit (Promega).

Experiments were performed independently at least three times (each sample measured in triplicate in each experiment) with consistent results.

For experimental validation of miRNA target sites using a luciferase reporter system, HEK293 cells were transfected with PEI and HL-1 cells were transfected with Lipofectamine 2000 (Invitrogen) according to manufacture instructions. Both cell lines were co-transfected with wild type or mutant *SHOX2* 3′UTR reporter constructs (200 ng) together with *mir*Vana miRNA mimics and/or *miR*Vana miRNA inhibitors at a final concentration of 5 nM. Double-stranded RNA designed to mimic the endogenous precursor *hsa*-*miR*-*92b*-*5p* as well as single-stranded RNA-based oligonucleotides designed to specifically bind to and inhibit *miR*-*92b*-*5p* and negative control miRNAs (Pre-miR miRNA Precursor Negative Control #1 and *miR*Vana miRNA Inhibitor Negative Control #1), were purchased from Ambion (Life Technologies). 48 h after transfection, luciferase activity was determined and normalized to Firefly luciferase activity with a dual luciferase assay kit (Promega). Experiments were performed independently at least three times (each sample measured in triplicate in each experiment) with consistent results.

### Zebrafish embryos and microinjections

Care and breeding of zebrafish, *Danio rerio*, were as described previously [51]. For all plasmid and morpholino injection procedures, the TE4/6 wild type strain was used. Morpholino-modified antisense oligonucleotides (MO; Gene Tools) were directed against the splice donor site of *shox2* intron 3, causing the identical bradycardia phenotype as the *shox2* MO directed against the translational start site. Both MOs have been described previously [6, 24]. *Shox2* antisense oligonucleotides or a standard control oligonucleotide (MO control), diluted in 0.2 mol/l KCl, were microinjected into one-cell-stage zebrafish embryos [27]. For rescue experiments 0.32 ng of *pDestTol2CG2*-*shox2 or pDestTol2CG2*-*shox2H227Q* was microinjected into one-cell-stage embryos directly after injection of 2.8 ng of *shox2* MO.

### Animals and tissue samples

CD-1 wild type mice were obtained from Janvier. Right atria of E11.5 hearts that comprise the *Shox2* expressing sinus venosus myocardium and venous valves and E11.5 total hearts were dissected. For total RNA isolation, TRIzol was used according to manufacturer’s instructions (Invitrogen).

### Human RNA samples

RNAs from human adult tissues were purchased from Ambion Life Technologies (FirstChoice Human Total RNA Survey Panel). The MRC-Wellcome Trust Human Developmental Biology Resource (HDBR) in Newcastle, England kindly provided RNAs from human fetal tissues.

### Quantitative PCR (qPCR)

cDNA was synthesized using the SuperScript First-Strand Synthesis System for RT-PCR (Invitrogen). For mRNA measurements, Hypoxanthine phosphoribosyl transferase 1 (Hprt1/HPRT1) and Succinate dehydrogenase (Sdha/SDHA) were used as housekeeping genes. MiRNA cDNA was synthesized using the MystiCq microRNA cDNA Synthesis Mix (Sigma-Aldrich). MystiCq microRNA qPCR Assay System with miRNA qPCR assay primers designed to target human and mouse *miR*-*92b*-*5p*, as well as human and mouse positive control primers (SNORD44 human small nucleolar and RNU6-1 mouse small nuclear RNAs, both ubiquitously expressed were used as housekeeping genes for miRNA qPCR) in combination with a MystiCq universal PCR primer were purchased from Sigma-Aldrich. Primer sets used are presented in Table S3. qPCR was performed on a 7500 Fast Real-Time PCR System (Applied Biosystems) using SYBR Green ROX dye (Thermo Scientific).

### miRNA measurement in human plasma

#### Plasma collection

Blood of all subjects was collected via a direct venous puncture into 9 ml EDTA tubes (Sarstedt Monovette). All blood was processed for isolation of plasma within 4 h of collection. Blood was processed by spinning at 4000 rpm for 20 min at RT. Plasma was carefully transferred to a fresh RNAse/DNAse free tube and stored at −80 °C.

#### RNA isolation and miRNA measurement

RNA isolation and miRNA measurement was described in detail previously [15]. In brief, total RNA was isolated from 400 ml EDTA-plasma using the miRNeasy kit (Qiagen). As no housekeeping miRNA has been established and validated to normalize for the miRNA content so far, we chose to use a fixed volume of plasma per sample and a synthetic Caenorhabditis elegans miR-39 (cel-miR-39, 20 fmol/sample, synthesized by Qiagen) as a spiked-in control to normalize for individual RNA-isolation-related variations. 20fmol cel-miR-39 were introduced to each plasma sample after addition of denaturating Qiazol solution. We used a fixed volume of 5 ml diluted RNA for reverse transcription using the TaqMan microRNA Reverse Transcription kit (Applied Biosystems) according to the manufacturer’s protocol. Subsequently, 1.33 μl of the product was used to detect miRNA expression by quantitative PCR using TaqMan qRT-PCR assays (Applied Biosystems). Primer sets used are presented in Table S3. Quantitative PCR reactions were performed on a Bio-Rad iQ5 system using the following program: 10 min pre-incubation at 95 °C, 40 cycles of 15 s denaturation at 95 °C and 60 s of elongation at 60 °C. Values are normalized to cel-miR-39 and expressed as 2^−[(CT microRNA)−(CT cel−miR−39)]^.

### Statistical analysis

For the clinical characteristics analysis and miRNA measurements in human plasma, data are presented as mean ± SEM. An unpaired student’s *t* test was used for two-group comparisons. Categorical variables were analyzed by Fisher’s exact test. For the association study a Chi squared (*χ*^2^) test was used. A *p* value *p* < 0.05 was considered statistically significant. For luciferase assays and zebrafish injections, data are presented as mean ± SEM from at least 3-4 independent experiments. Comparisons between experimental groups were performed with GraphPad Prism 6 using paired *t* test or 1-way ANOVA. Differences were considered significant if they showed a *p*-value of *p* < 0.05.

## Electronic supplementary material

Below is the link to the electronic supplementary material.

Supplementary material 1 (DOCX 376 kb)
