# Supplementary material for: Coding and non-coding variants in the SHOX2 gene in patients with early-onset atrial fibrillation
Source: Basic Res Cardiol. 2016 Apr 30;111:36. doi: 10.1007/s00395-016-0557-2 (PMC4853439; doi:10.1007/s00395-016-0557-2)
Supplement: Supplementary file 1 — Supplementary material 1 (DOCX 376 kb) [file 395_2016_557_MOESM1_ESM.docx]

**Supplemental Data**

**Coding and non-coding variants in the *SHOX2* gene in patients with early-onset atrial fibrillation**

Sandra Hoffmann; Sebastian Clauss; Ina M. Berger; Birgit Weiß; Antonino Montalbano; Ralph Röth; Madeline Bucher; Ina Klier; Reza Wakili; Hervé Seitz; Eric Schulze-Bahr; Hugo A. Katus; Friederike Flachsbart; Almut Nebel; Sabina PW. Guenther; Erik Bagaev; Wolfgang Rottbauer; Stefan Kääb; Steffen Just and Gudrun A. Rappold

**
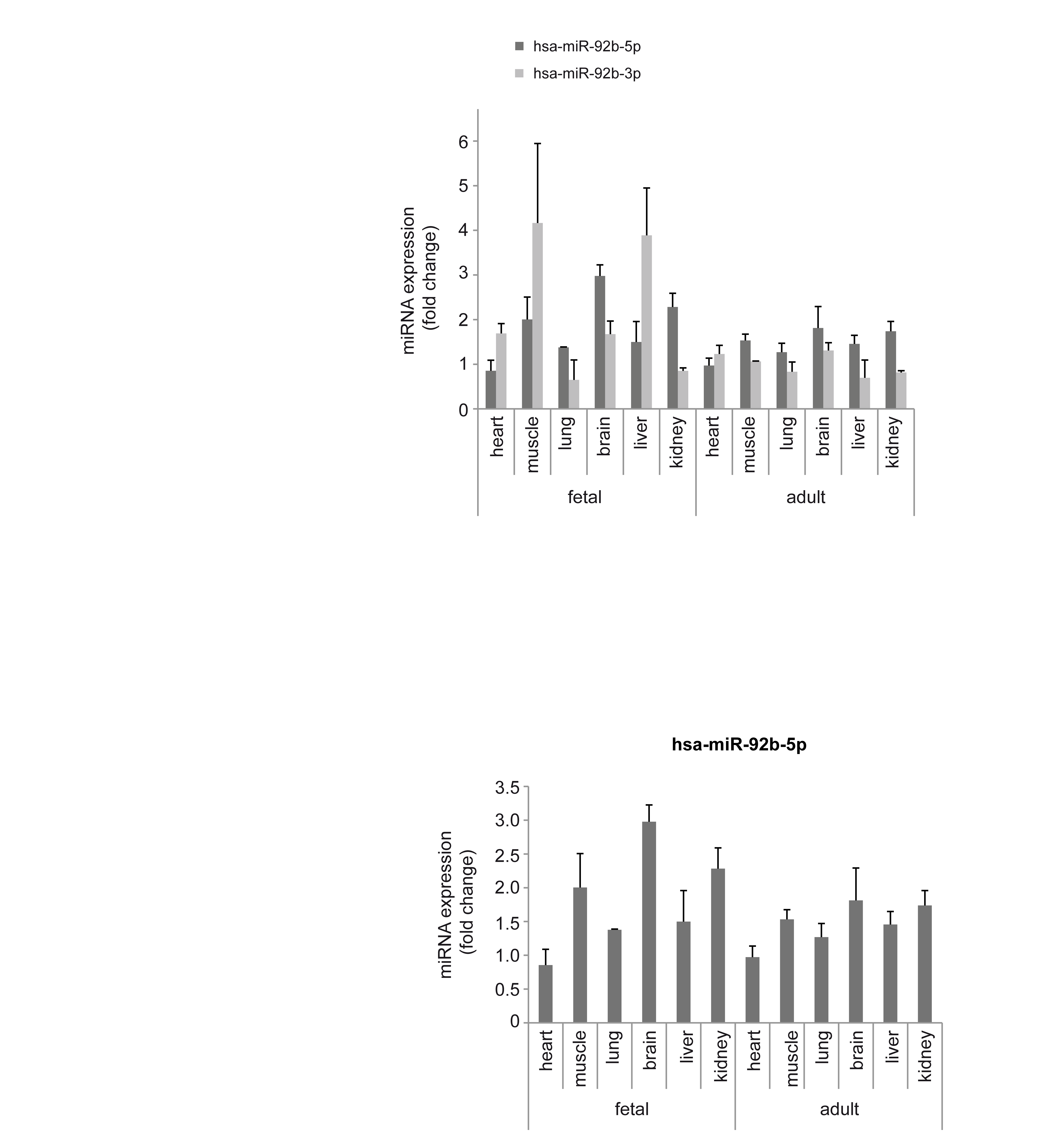
**

**Fig. S1. *Hsa-miR-92b-5p* expression analysis.**

*miR-92b-5p* is ubiquitously expressed in various human fetal and adult tissues. Measurements were performed in duplicates.

**
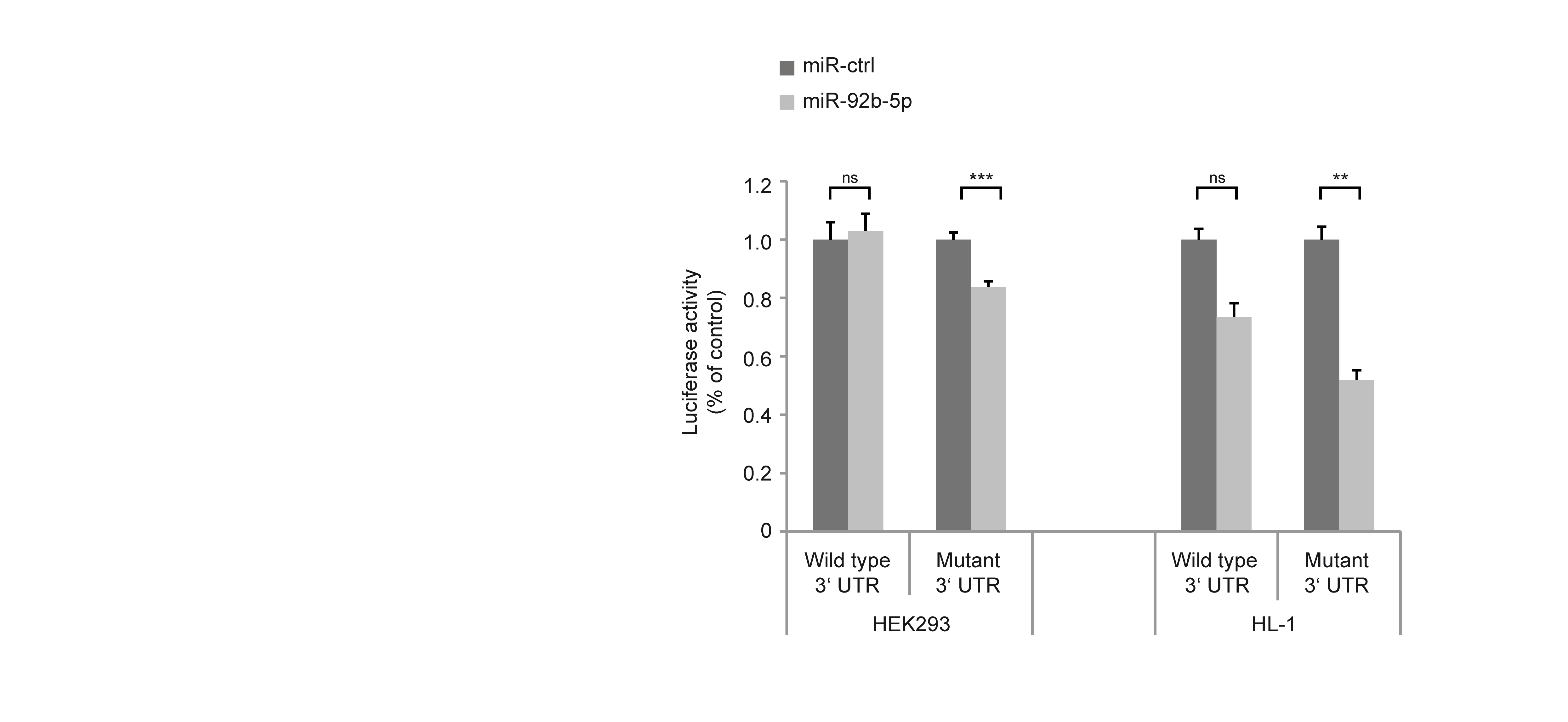
**

**Fig. S2. Allele-specific effects of *miR-92b-5p* on luciferase activity by mutant pRL-TK-*SHOX2* 3’UTR c.*28C reporter using part of the 3’UTR.**

Relative luciferase activity of wild type (c.*28T) or mutant (c.*28C) pRL-TK-*SHOX2* 3’UTR constructs harboring 977bp (48%) of the 3’UTR, co-expressed with *miR-92b-5p* (indicated in light grey) or negative control miRNA (miR-ctrl, indicated in dark grey) in HEK293 and HL-1 cells, determined 48h after transfection. *miR-92b-5p* significantly reduces the activity of the mutant but not the wild type *SHOX2* 3’UTR reporter in both cell lines. Data are expressed as means ±SEM of 6 independent experiments. For each experiment triplicates were measured. *p*-values were determined by a paired *t* test (***p*<0.01; ****p*<0.001, ns=not significant).

| **Clinical characteristics** | **AF patients with**  **T/T genotype** | **AF patients with**  **T/C genotype** | ***p*-value** |
| --- | --- | --- | --- |
|  |  |  |  |
| **Demographic parameters** | | | |
| Age at onset, (y) | 47.1±0.5 | 49.6±2.4 | 0.912 |
| Male gender, n (%) | 266/356 (74.7%) | 9/15 (60.0%) | 0.230 |
|  |  |  |  |
| **Atrial Fibrillation** | | | |
| Paroxysmal AF, n (%) | 216/340 (63.5%) | 7/14 (50.0%) | 0.398 |
|  |  |  |  |
| **Family History** | | | |
| Atrial fibrillation, n (%) | 67/342 (19.6%) | 4/15 (26.7%) | 0.510 |
| Cardiovascular diseases, n (%) | 81/353 (23.0%) | 5/14 (35.7%) | 0.331 |
|  |  |  |  |
| **Echocardiography** | | | |
| Ejection fraction, (%) | 58.6±1.1 | 47.8±9.1 | 0.203 |
| LA diameter, (mm) | 47.6±1.7 | 47.4±3.3 | 0.977 |
|  |  |  |  |
| **ECG parameters** | | | |
| **PR interval, (msec.)** | **173.1±1.9** | **216.7±16.1** | **0.0002** |
| RR interval, (msec.) | 848.9±12.7 | 803.3±63.0 | 0.488 |
| QRS duration, (msec.) | 98.0±3.9 | 86.0±5.2 | 0.576 |
| QT interval, (msec.) | 391.6±4.9 | 370.0±17.5 | 0.413 |
|  |  |  |  |
| **Concomitant diseases** | | | |
| Coronary heart disease, n (%) | 41/344 (11.9%) | 2/15 (13.3%) | 0.698 |
| History of MI, n (%) | 17/348 (4.9%) | 1/14 (7.1%) | 0.517 |
| Valvular heart disease (> II°), n (%) | 22/350 (6.3%) | 0/14 (0.0%) | 1.000 |
| DCM, n (%) | 31/348 (8.9%) | 1/14 (7.1%) | 1.000 |
| Hyperthyroidism, n (%) | 13/350 (3.7%) | 1/14 (7.1%) | 0.428 |
|  |  |  |  |
| **Cardiovascular risk factors** | | | |
| Hypertension, n (%) | 175/355 (49.3%) | 8/15 (53.3%) | 0.798 |
| Diabetes mellitus, n (%) | 35/355 (9.9%) | 3/14 (21.4%) | 0.165 |
| Hypercholesterolemia, n (%) | 83/354 (23.5%) | 3/14 (21.4%) | 1.000 |
|  |  |  |  |

**Table S1**. **Clinical characteristics of 378 patients with early-onset atrial fibrillation.**

Overview on demographic data, AF type, family history, echocardiography, ECG parameters, concomitant diseases, and cardiovascular risk factors in the patient cohort evaluated. Abbreviations: AF=Atrial fibrillation; LA=Left atrium; ECG= Electrocardiographic; MI=Myocardial infarction; DCM=Dilated cardiomyopathy.

| **Clinical characteristics** | **AF patients with**  **T/T genotype** | **AF patients with**  **T/C genotype** | ***p*-value** |
| --- | --- | --- | --- |
|  |  |  |  |
| **Demographic parameters** | | | |
| Age at onset, (y) | 51.3±1.6 | 55.4±1.4 | 0.107 |
| Male gender, n (%) | 11/17 (64.7%) | 2/6 (33.3%) | 0.341 |
|  |  |  |  |
| **Atrial Fibrillation** | | | |
| Paroxysmal AF, n (%) | 7/17 (41.2%) | 2/6 (33.3%) | 1.000 |
|  |  |  |  |
| **Family History** | | | |
| Atrial fibrillation, n (%) | 3/17 (17.6%) | 2/6 (33.3%) | 0.576 |
| Cardiovascular diseases, n (%) | 5/17 (29.4%) | 0/6 (0.0%) | 0.273 |
|  |  |  |  |
| **Echocardiography** | | | |
| Ejection fraction, (%) | 60.2±2.8 | 55.5±0.05 | 0.780 |
| LA diameter, (mm) | 44.3±1.5 | 43.5±3.9 | 0.831 |
|  |  |  |  |
| **ECG parameters** | | | |
| PR interval, (msec.) | 184.4±9.8 | 203.3±12.0 | 0.434 |
| RR interval, (msec.) | 894.1±28.9 | 803.0±92.8 | 0.221 |
| QRS duration, (msec.) | 94.6±4.8 | 88±5.8 | 0.485 |
| QT interval, (msec.) | 425.6±8.2 | 419±12.0 | 0.682 |
|  |  |  |  |
| **Concomitant diseases** | | | |
| Coronary heart disease, n (%) | 2/17 (11.8%) | 1/6 (16.7%) | 1.000 |
| History of MI, n (%) | 0/17 (0.0%) | 0/6 (0.0%) |  |
| Valvular heart disease (> II°), n (%) | 1/17 (5.9%) | 0/6 (0.0%) | 1.000 |
| DCM, n (%) | 2/17 (11.8%) | 0/6 (0.0%) | 1.000 |
| Hyperthyroidism, n (%) | 0/17 (0.0%) | 1/6 (16.7%) | 0.292 |
|  |  |  |  |
| **Cardiovascular risk factors** | | | |
| Hypertension, n (%) | 10/17 (58.8%) | 3/6 (50.0%) | 1.000 |
| Diabetes mellitus, n (%) | 2/17 (11.8%) | 1/6 (16.7%) | 1.000 |
| Hypercholesterolemia, n (%) | 9/17 (52.9%) | 1/6 (16.7%) | 0.179 |
|  |  |  |  |

**Table S2**. **Clinical characteristics of patient cohort evaluated for miRNA plasma expression.**

Overview on demographic data, AF type, family history, echocardiography, ECG parameters, concomitant diseases, and cardiovascular risk factors in the patient cohort evaluated. Abbreviations: AF=Atrial fibrillation; LA=Left atrium; ECG= Electrocardiographic; MI=Myocardial infarction; DCM=Dilated cardiomyopathy.

| **Table S3.** Oligonucleotides | | | | |
| --- | --- | --- | --- | --- |
| **Name** | **Sequence (5' - 3')** | **T_M_ (°C)** | **Product size (bp)** | **Application** |
| SHOX2 Ex1.1_5’UTR for | TGT AAA ACG ACG GCC AGT AGA GGT TGA GCG CCG GGC TGA CGT | 62 | 269 | **Sequencing** |
| SHOX2 Ex1.1_5’UTR rev | GGA TAA CAA TTT CAC ACA GGC CTA CAC CTC CTC CGC CTC CTC CG |  |  |  |
| SHOX2 Ex 1.2 for | TGT AAA ACG ACG GCC AGT CGC AGC AGC CCG GCA GTC CGG GC | 58 | 222 |  |
| SHOX2 Ex 1.2 rev | GGA TAA CAA TTT CAC ACA GGC TGC CGG GGG TCA GTC AGG TCG T |  |  |  |
| SHOX2 Ex2+ for | CCG AGT ACT GGG TGA TTG | 57 | 239 |  |
| SHOX2 Ex2+ rev | GCC AAG ACC CCT CGA ACT |  |  |  |
| SHOX2 Ex2 for | TCC ACG AGG GGG AAG GAT TC | 62 | 458 |  |
| SHOX2 Ex2 rev | CAC CAG ACA CTA GAA GCA CCA |  |  |  |
| SHOX2 Ex3 for | TGT AAA ACG ACG GCC AGT TTG CTT GCT GTA TCT CCC AAT | 59 | 223 |  |
| SHOX2 Ex3 rev | GGA TAA CAA TTT CAC ACA GGT TTG CTC AGA CTA TCA AAT GTT CC |  |  |  |
| SHOX2 Ex4 for | TGT AAA ACG ACG GCC AGT TTT GGA ACC CTG AAA AAT GC | 58 | 242 |  |
| SHOX2 Ex4 rev | GGA TAA CAA TTT CAC ACA GGG GCT CAG AGA CAG GTG ATG |  |  |  |
| SHOX2 Ex5 for | TGT AAA ACG ACG GCC AGT CCC AAA CAC AAC CCA ACT CT | 60 | 211 |  |
| SHOX2 Ex5 rev | GGA TAA CAA TTT CAC ACA GGG CTG GGA ACA TCA TGT AGG G |  |  |  |
| SHOX2 Ex6_3’UTR for | AGG ATA GTC ATT GCA ACG TGA | 60 | 387 |  |
| SHOX2 Ex6_3’UTR rev | TCT CAA AGG GGT AAC GGA GA |  |  |  |
| mHprt1 qRT for | TCC TCC TCA GAC CGC TTT T | 60 | 90 | **qRT-PCR** |
| mHprt1 qRT rev | CCT GGT TCA TCA TCG CTA ATC |  |  |  |
| mSdha qRT for | Cat gcc agg gaa gat tac aaa | 60 | 88 |  |
| mSdha qRT rev | Gtt ccc caa acg gct tct |  |  |  |
| mShox2 qRT for | ACC AAT TTT ACC CTG GAA CAA C | 60 | 141 |  |
| mShox2 qRT rev | TCG ATT TTG AAA CCA AAC CTG |  |  |  |
| hHPRT1 qRT for | TGA TAG ATC CAT TCC TAT GAC TGT AGA | 60 | 126 |  |
| hHPRT1 qRT rev | AAG ACA TTC TTT CCA GTT AAA GTT GAG |  |  |  |
| hSDHA qRT for | TGG GAA CAA GAG GGC ATC TG | 60 | 86 |  |
| hSDHA qRT rev | CCA CCA CTG CAT CAA ATT CAT G |  |  |  |
| hSHOX2 qRT for | GAC CAA AAT CAA GCA GAG GCG AAG T | 60 | 298 |  |
| hSHOX2 qRT rev | CTG CTG AAA TGG CAT CCT TAA AGC ACC |  |  |  |
| SHOX2 3’UTR_AsiSI for | TAT ATg cga tcg ccg cca acg cca gca cca atg t | 50 | 2015 | **Cloning** |
| SHOX2 3’UTR_NotI rev | TAg cgg ccg cCA AAT GAA ACG ATA TTT TCA TTC AAT T |  |  |  |
| SHOX2 3’UTR_Xba I for | GGG TCT AGA CGC CAA CGC CAG CAC CAA TGT | 50 | 978 |  |
| SHOX2 3’UTR_XbaI rev | CCC TCT AGA CAA ATG AAA CGA TAT TTT CAT TCA ATT |  |  |  |
| SHOX2_G81E for | ggt gta gga gga gaa gga gca ggc gga |  | | **Mutagenesis** |
| SHOX2_G81E rev | tcc gcc tgc tcc ttc tcc tcc tac acc |  |  |  |
| SHOX2_H283Q for | aca gcg ctg tgg cgc aag cgc acc ac |  |  |  |
| SHOX2_H283Q rev | gtg gtg cgc ttg cgc cac agc gct gt |  |  |  |
| SHOX2_3’UTR Mut for | cac caa tgt cgc gcc cgt ccc gcg gca ctc |  |  |  |
| SHOX2_3’UTR Mut rev | gag tgc cgc ggg acg ggc gcg aca ttg gtg |  |  |  |
| shox2_H227Q for | CAG CGC CGT GGC TCA aGC GCA TCA TCA CCT G |  |  |  |
| shox2_H227Q rev | CAG GTG ATG ATG CGC tTG AGC CAC GGC GCT G |  |  |  |
| **Name** | **Target sequence (5' - 3')** | **Assay ID** | **miR Base Accession** | **Application** |
| Hsa-miR-92b-5p | agg gac ggg acg cgg ugc agu g | 002343 | MIMAT0004792 | **miRNA qPCR** |
| Cel-miR-39 | UCA CCG GGU GUA AAU CAG CUU G | 000200 | MI0000010 |  |
| Hsa-miR-92b-5p | agg gac ggg acg cgg ugc agu g | MIRAP00107 | MIMAT0004792 |  |
| Mmu-miR-92b-5p | AGG GAC GGG ACG UGG UGC AGU GUU | MIRAP01237 | MIMAT0017278 |  |
| Hsa-miR-92b-3p | UAU UGC ACU CGU CCC GGC CUC C | MIRAP00106 | MIMAT0003218 |  |
| SNORD44 control | CCU GGA UGA UGA UAA GCA AAU GCU GAC UGA ACA UGA AGG UCU UAA UUA GCU CUA ACU GAC U | MIRCP00005 | - |  |
| RNU6-1 control | GUG CUC GCU UCG GCA GCA CAU AUA CUA AAA UUG GAA CGA UAC AGA GAA GAU UAG CAU GGC CCC UGC GCA AGG AUG ACA CGC AAA UUC GUG AAG CGU UCC AUA UUU U | MIRCP00001 | - |  |
| SHOX2  GAPDH |  | Hs00243203 m1  Hs02758991 g1 | 129  93 | **qPCR** |
